# Preparation of water-soluble nanographite and its application in water-based cutting fluid

**DOI:** 10.1186/1556-276X-8-52

**Published:** 2013-01-26

**Authors:** Qiang Chen, Xue Wang, Zongting Wang, Yu Liu, Tingzheng You

**Affiliations:** 1 State Key Laboratory of Heavy Oil Processing, China University of Petroleum (East China), Qingdao, 266580, People’s Republic of China; 2 College of Science, China University of Petroleum (East China), Qingdao, 266580, People’s Republic of China

**Keywords:** Nanographite, Emulsion polymerization, Lubrication performance, Water-based cutting fluid

## Abstract

Water-soluble nanographite was prepared by *in situ* emulsion polymerization using methacrylate as polymeric monomer. The dispersion stability and dispersion state of graphite particles were evaluated by UV-visible spectrophotometry and scanning electron microscopy, respectively. The water-soluble nanographite was then added into the water-based cutting fluid as lubricant additive. The lubrication performance of water-based cutting fluid with the nanographite additive was studied on four-ball friction tester and surface tensiometer. Results indicate that the modification method of *in situ* emulsion polymerization realizes the uniform and stabilized dispersion of nanographite in aqueous environment. The optimal polymerization condition is 70°C (polymerization temperature) and 5 h (polymerization time). The addition of nanographite decreases the friction coefficient and wear scar diameter by 44% and 49%. Meanwhile, the maximum non-seizure load (*P*_*B*_) increases from 784 to 883 N, and the value of surface tension (32.76 × 10^−3^ N/m) is at low level. Nanographite additive improves apparently the lubrication performance of water-based cutting fluid.

## Background

In recent years, nanographite has received considerable attention due to its natural features [1]. On one hand, nanographite possesses the special properties of nanomaterials such as the quantum-size effect, the small-size effect, and the surface or interface effect [2]. On the other hand, it has the advantages of natural graphite flakes such as the self-lubrication and boundary-lubrication abilities. Therefore, nanographite exhibits great superiority in the lubrication field, especially under harsh circumstances like high-temperature or extreme-pressure conditions [3,4]. However, nanographite is difficult to apply in water-based fluid because of its hydrophobicity [5-7].

Cutting fluid plays an important role in the manufacturing industry as lubricant [8]. It can be mainly classified into two categories: oil-based and water-based cutting fluid. The primary functions of cutting fluid include lubrication, cooling, cleaning, and antirust. At present, the lubrication performance of oil-based cutting fluid is outstanding, but its cooling property is inferior. On the contrary, water-based cutting fluid shows powerful ability in cooling, cleaning, and antirust, but it is relatively weak in lubrication [9]. Nowadays, increasingly strict environmental regulations result in higher operating costs for metal cutting. Water-based cutting fluid is utilized more and more popularly, owing to its low-cost and less-waste emissions than oil-based cutting fluid [10]. However, the water-based cutting fluid is not ideal due to its inferior lubrication ability [8]. Consequently, it is necessary to find a way to enhance the lubrication property of water-based cutting fluid. Up to now, a great deal of research has been done on this subject [9-11]. One simple approach is putting additives into regular lubricants to reduce friction and wear, which has been widely applied in lubrication engineering [2].

Many researchers [12-14] have reported that nanoadditives are effective in improving the properties of lubricants. They applied different kinds of nanoparticles made of polymer, metal, organic, or inorganic materials to the fabrication of nanolubricants. In order to make the sufficient exertion of the lubricating advantage of nanographite, this research aims to improve the lubrication performance of water-based cutting fluid by adding nanographite as an additive [15]. In this study, commercially available nanographite and water-based cutting fluid were used as materials. Graphite nanoparticles were firstly modified through *in situ* emulsion polymerization to obtain the water-soluble nanographite [16-19]. UV-visible (vis) spectrophotometry was used to evaluate dispersion stability and determine the optimal polymerization condition. Afterwards, water-soluble nanographite was added into water-based cutting fluid as lubrication additive. The dispersion state of nanographite [20] in aqueous environment was characterized by scanning electron microscopy (SEM), and the lubrication performance of water-based cutting fluid with nanographite additive was tested by some tribological experiments.

## Methods

### Materials

**Figure 1:**
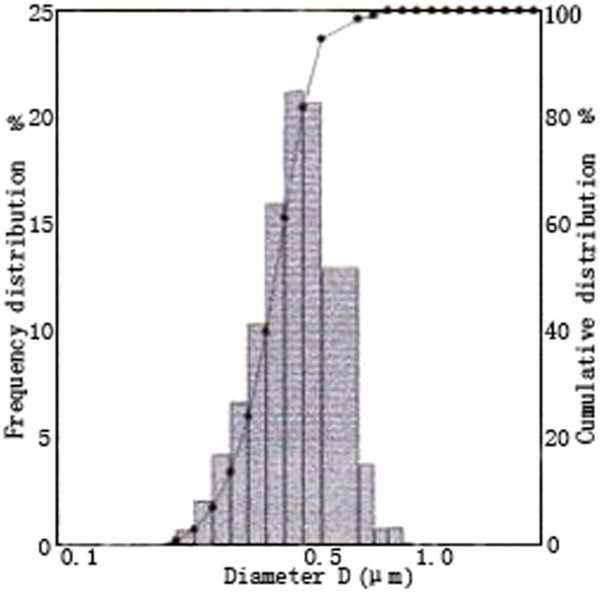
Particle size distribution of nanographite.

Commercially available nanographite (Qingdao HuaTai Lubricant Co., Qingdao, China; D50 = 400 nm) was used in the research. The size distribution of the graphite nanoparticles is shown in Figure 1. Commercially supplied water-based cutting fluid (Qingdao Tang Qi Lubrication Technology Co., Ltd. Qingdao, China; QDW618) was used as the base fluid. Its basic properties are listed in Table 1. In the table, GB/T6144 is the Chinese National Standard test methods of synthetic cutting fluids. Test methods of different properties are as follows:

1. pH: immerse pH test strip into the test solution, and then contrast it with the standard strip.

2. Foam volume: pour the test solution (70 mL) into a 100-mL cylinder with a stopper. After shaking (1 min) and stewing (10 min), observe the volume of the remaining foam.

3. Surface tension: test using an interface tensiometer.

4. Antirust ability: measure by cast iron (two categories, single or lamination). GB/T3142 is the Chinese National Standard test methods of lubricants (determination of load-carrying capacity). Both maximum non-seizure load (*P*_*B*_) and weld load (*P*_*D*_) are tested on a four-ball friction tester.

**Table 1:** Basic properties of QDW618 water-based cutting fluid

**Property**	**pH**	**Foam volume *V *(ml) (≯)**	**Surface tension *σ *(mN/m) (≯)**	**Antirust ability *t *(h)**		**Abrasion resistance *f *(N) (≮)**	
				**Single**	**Lamination**	***P***_***B***_	***P***_***D***_
Value	8 ~ 10	2	40	24	4	800	2300
Method	GB/T6144/5.3	GB/T6144/5.4	GB/T6144/5.7	GB/T6144/5.7		GB/T3142	

### Preparation of water-soluble nanographite

The hydrophobicity of graphite nanoparticles is the major impediment in using nanographite as an additive in water-based fluid to improve the lubrication performance. In order to take the lubrication advantage of nanographite to water-based fluid, surface modification is necessitated to obtain water-soluble nanographite. In this study, water-soluble nanographite was prepared through *in situ* emulsion polymerization using methacrylate as polymeric monomer. Prior to polymerization reaction, graphite nanoparticles were pretreated by ultrasonic dispersion. The nanographite (1.0 wt.%) was added into a water solution with sodium dodecyl benzene sulfonate (SDBS). As surfactant, SDBS could favor the dispersion of graphite nanoparticles during the ultrasonic process. Ultrasonic pretreatment was carried on an ultrasonic treatment device (Shanghai Ultrasonic Device Co., Shanghai, China; FS-250) for 10 min. The effects of ultrasonic dispersion were observed by SEM. Methacrylate was refined by vacuum distillation before being used as polymeric monomer. The refined methacrylate and the pretreated nanographite were mixed into a four-necked flask. Three of the four necks were used to connect the thermometer, stirring device, and nitrogen, respectively. The other one was left for sampling. A spot of sodium bicarbonate (0.1 wt.%) was also added into the mixture to adjust the pH. Potassium persulfate was employed as the initiator of polymerization. The reaction temperatures were set as 60°C, 70°C, and 80°C. Under each reaction temperature, the sampling time was 4, 5, and 6 h. The entire experiment was conducted under nitrogen atmosphere. The final samples were separated by centrifugation (3,000 rpm, 30 min), and the supernatants were collected.

### Characterization

Absorbance of different supernatants was measured by UV–vis spectrophotometry (Shimadzu Co., Nakagyo-ku, Kyoto, Japan; UV-2450) to evaluate the dispersion stability. The spectral region is 700 to approximately 250 nm. In the experiment, one of the colorimetric wares was enclosed by the supernatant with nanographite as testing sample, and the other one was enclosed by the supernatant without nanographite as reference sample. The dispersion state of graphite particles in aqueous environment was characterized by SEM (Hitachi High-Tech, Minato-ku, Tokyo, Japan; S-4800). SEM images under different magnifications displayed the micromorphology of graphite emulsion.

### Tribological tests

The supernatant (obtained under optimal polymerization condition) was added into QDW618 water-based cutting fluid with the ratio of 2.0 wt.%. This mixture was named as nanographite fluid. The QDW618 water-based cutting fluid had been diluted by deionized water with the ratio of 1:10. The diluted QDW618 was named as base fluid to make contrast with the nanographite fluid. A series of tribological parameters were obtained by the four-ball friction tester (Jinan Co., Jinan, China; MR-10A) to evaluate the lubrication performance of the nanographite fluid and base fluid. Conditions of the four-ball wear tests are 600 rpm (spindle speed), 392 N (loads), and 1 h (testing time). Also, the frictional materials in the tests were GCr15 standard steel balls. The maximum non-seizure load (*P*_*B*_) was measured according to GB3142-82 (Chinese National Standard: spindle speed 1,400 to approximately 1,500 rpm, testing time 10 s). In addition, the surface tension was tested on a surface tensiometer (Kruss Co., DKSH Hong Kong Limited, Shanghai, China; K-12) to investigate the wettability.

## Results and discussion

### Effects of ultrasonic dispersion

**Figure 2:**
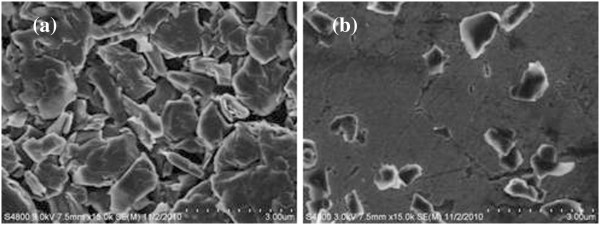
**Effects of ultrasonic pretreatment on graphite particles. **(**a**) Before ultrasonic pretreatment and (**b**) after ultrasonic pretreatment.

The effects of ultrasonic dispersion can be observed in the SEM images (Figure 2). Figure 2a displays the state of graphite particles before ultrasonic pretreatment. It can be seen that the graphite particles are in agglomeration and that the size distribution is uneven. As shown in Figure 2b, the aggregates are broken down, and the particle size reduces distinctly after ultrasonic dispersion. The graphite particles realize the preliminary dispersion via ultrasonic pretreatment. This will certainly favor the following modification. Therefore, it is a significant procedure to do ultrasonic dispersion before emulsion polymerization. However, this kind of dispersion is unstable because it does not change the surface properties of graphite particles.

### Dispersion stability

**Figure 3:**
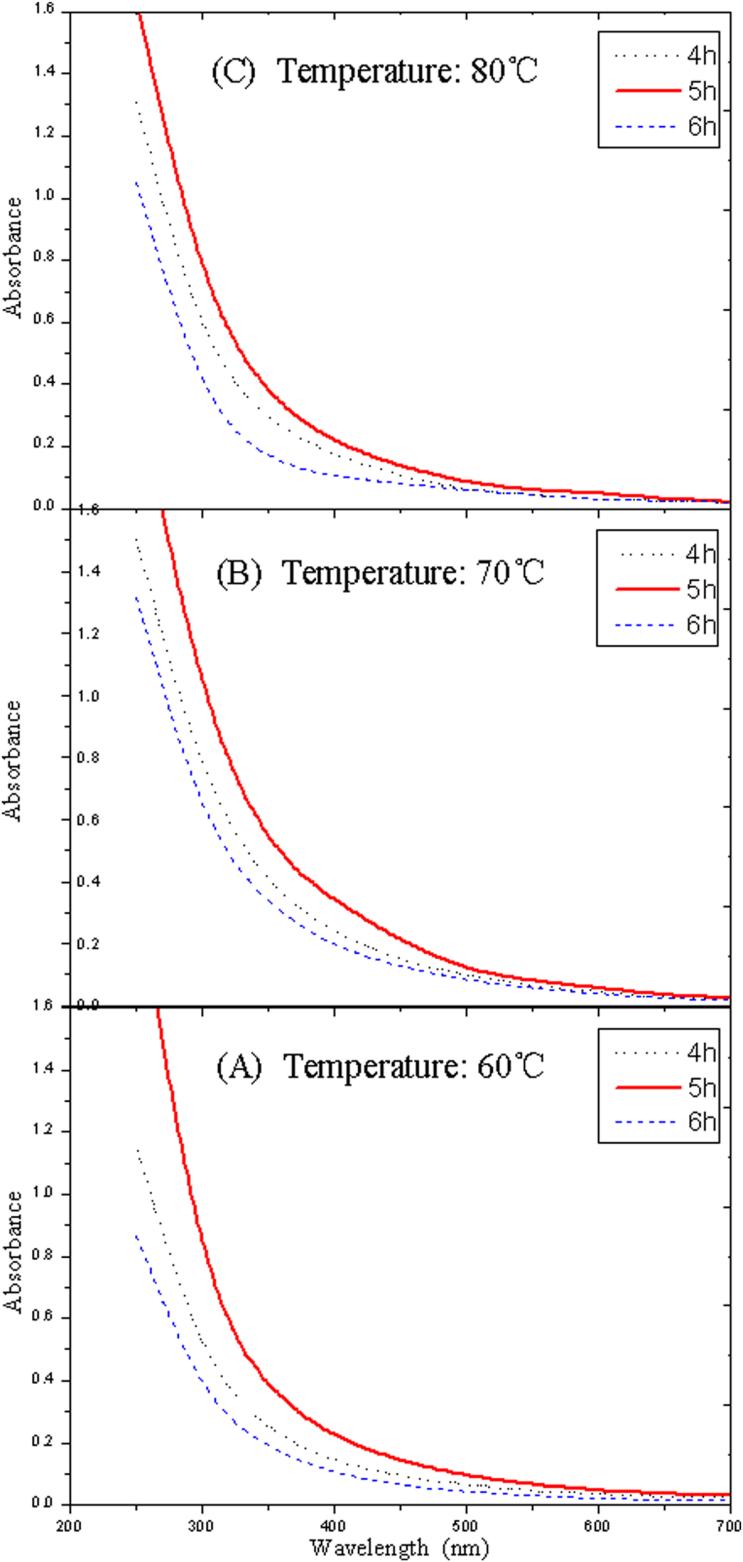
**Change of absorbance with wavelength under different polymerization conditions.** Temperatures at (**A**) 60°C, (**B**) 70°C, and (**C**) 80°C.

**Figure 4:**
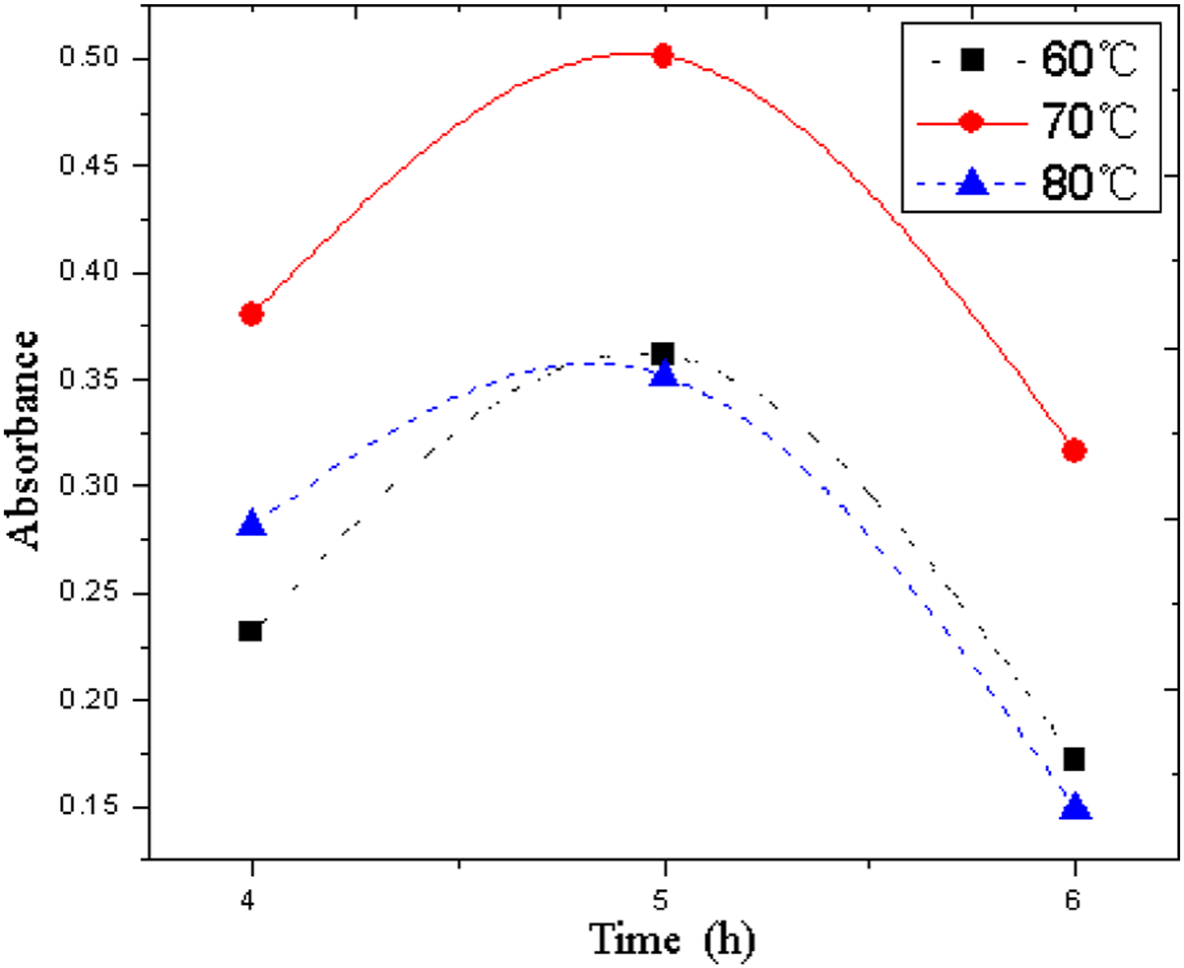
Absorbance under different polymerization conditions at the wavelength of 350 nm.

Water-soluble nanographite is prepared through *in situ* emulsion polymerization of methyl acrylate in the presence of nanographite. It is an important step that the emulsion is centrifuged after polymerization. On one hand, centrifugal separation could remove the graphite particles which do not dissolve in water. On the other hand, it has accelerated the settlement process of graphite emulsion so as to evaluate the dispersion stability. After centrifugation, the supernatants are separated to analyze the absorbance on a UV–vis spectrophotometer. Figure 3 shows the changing curves between absorbance and wavelength at different temperatures. The curves in Figure 3 exhibit a similar change tendency. There is nearly no absorption when the wavelength is beyond 550 nm. The absorbance increases with the decrease of wavelength in the range of 550 to approximately 250 nm, and the increasing rate becomes larger and larger. There exhibits a one-to-one correspondence between absorbance and wavelength within the range 550 to approximately 250 nm. Any wavelength in this range could be used as the characteristic absorption wavelength to evaluate the dispersion stability of graphite emulsion. In this study, 350 nm is selected as the fixed detection wavelength. Figure 4 displays the absorbance under different polymerization conditions at 350 nm. According to the Lambert-Beer law *A* = *εLc* (*A* absorbance; *ε* absorptivity; *L* width of colorimetric ware; *c* concentration), the absorbance is proportional to the concentration of graphite emulsion, and the concentration could then reflect the dispersion stability of graphite particles in the emulsion. From Figure 4, the maximum absorbance is corresponding to the condition of 70°C (polymerization temperature) and 5 h (polymerization time). Therefore, 70°C and 5 h is considered as the optimal polymerization condition. The water-soluble nanographite obtained under this condition is chosen to be the lubrication additive of water-based cutting fluid.

### Dispersion state

**Figure 5:**
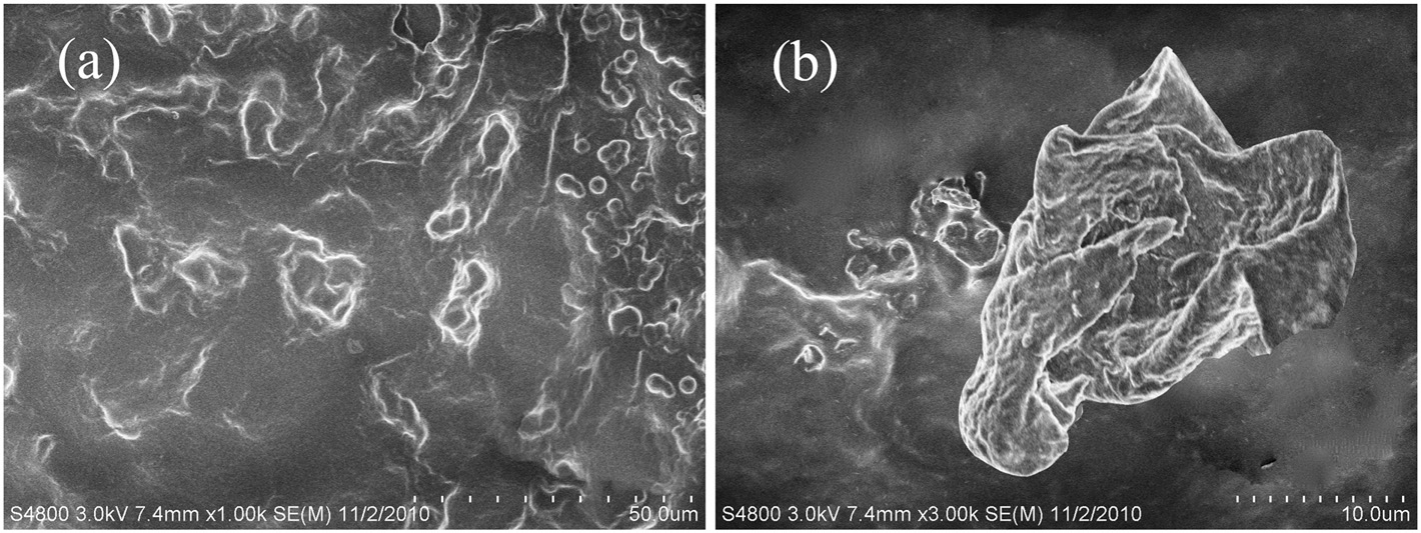
**Microdispersion state of graphite particles. **SEM images (**a**) ×1,000 and (**b**) ×3,000.

Figure 5 shows the microdispersion state of graphite particles in aqueous environment. Figure 5a,b shows SEM images with different magnifications. It can be indicated from Figure 5a that the graphite particles are uniformly dispersed in the emulsion. The agglomeration between graphite particles is avoided effectively. From Figure 5a, it could be recognized that there is a membrane-like substance coating around the graphite particles. This demonstrates that the nanographite/polymethyl acrylate composite is synthesized successfully. Figure 5b is the partial amplification image of Figure 5a. It displays the morphology of a single graphite flake which is coated by the polymethyl acrylate membrane. The surface of the graphite particle is modified by emulsion polymerization, and the original laminated structure of the nanographite is not destroyed. However, the particle size increases after emulsion polymerization.

**Figure 6:**
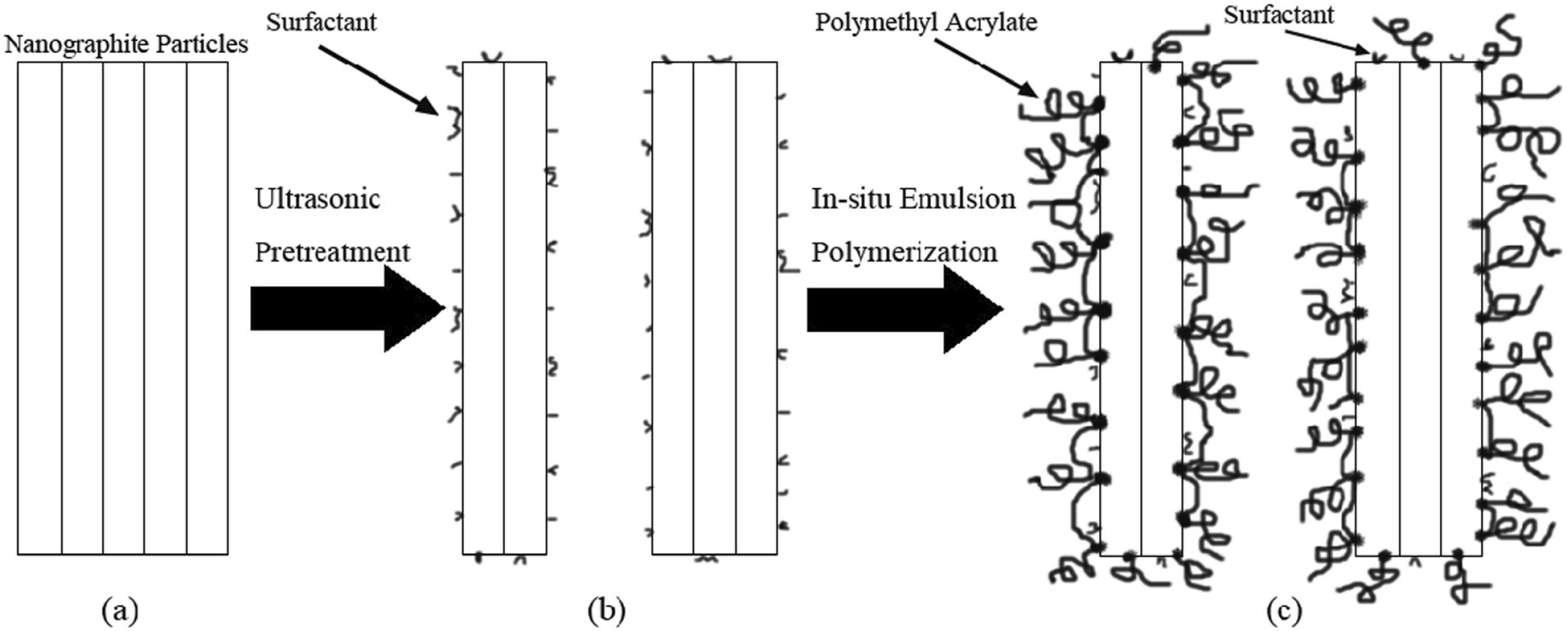
**Synthesis process and action mechanism of water-soluble nanographite. **(**a**) In agglomeration, (**b**) preliminary dispersion, and (**c**) stabilized dispersion.

Figure 6 is drawn to explain the synthesis process and action mechanism of water-soluble nanographite. The nanographite materials are in agglomeration at the beginning (Figure 6a). After ultrasonic pretreatment, the agglomerations are broken into small ones, and the surfactant adsorbs on the surface of small graphite particles. The nanographite realizes the preliminary dispersion at this stage (Figure 6b). Through *in situ* emulsion polymerization, the nanographite/polymethyl acrylate composite is synthesized as shown in Figure 6c. The surface of nanographite is completely covered and encapsulated by polymethyl acrylate. The hydrophobic moieties of polymethyl acrylate are embedded in the surface of nanographite particles, and the hydrophilic ones are dissolved in aqueous environment. The coating of polymethyl acrylate can reduce the interparticle force and produce steric hindrance which results in the reduced possibility of agglomeration of nanographite particles.

### Tribological properties

**Figure 7:**
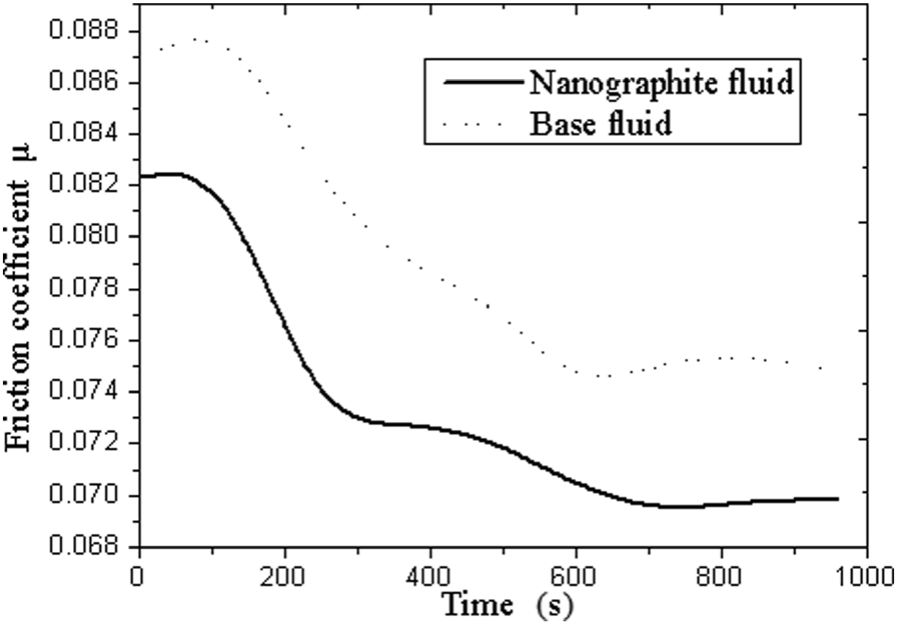
Relationship between the friction coefficient and testing time.

Tribological tests were conducted on the four-ball friction tester. Table 2 shows the basic parameters of base fluid and nanographite fluid. The friction coefficient is an important factor in evaluating the characteristics of lubricants. It could be concluded from Table 2 that the mean friction coefficient of nanographite fluid decreases by 44% in comparison with the base fluid. It demonstrates that the water-soluble nanographite plays a good lubricant role during the friction process. The relationship between the friction coefficient and testing time is shown in Figure 7. In general, the friction coefficient decreases over testing time, but it becomes stable after 800 s. Relatively speaking, the friction coefficient of the nanographite fluid is smaller than the base fluid at the same testing time. Meanwhile, wear scar diameter (WSD) decreases by 49% (from 1.27 to 0.65 mm), and *P*_*B*_ value increases from 784 to 883 N. These data indicate that the extreme pressure and antiwear properties of water-based cutting fluid improve prominently, owing to the addition of nanographite. There is a significant reduction in direct metal contact in the presence of nanographite particles. In addition, the surface tension of the nanographite fluid (32.76 × 10^−3^ N/m) is at low level. It increases the wettability of the cutting fluid and thereby helps the spreading on the surface of workpiece.

**Table 2:** Tribological parameters of base fluid and nanographite fluid

**Tribological parameters**	**Base fluid**^**a**^	**Nanographite fluid**^**b**^
Mean friction coefficient (μ)	0.106	0.059
WSD *D* (mm)	1.27	0.65
Maximum non-seizure load *P*_*B *_(N)	784	883
Surface tension *σ *(×10^−3^ N/m)	33.04	32.76

^a^Diluted QDW618 water-based cutting fluid; ^b^Diluted QDW618 water-based cutting fluid with nanographite additive.

The cutting fluid owes its lubrication ability from the lubricating film between the cutter and workpiece. Nanographite particles possess the features of high-temperature resistance and self-lubrication ability which favor the formation and strengthening of the lubricating film. Therefore, the nanographite additive improves apparently the lubrication performance of the water-based cutting fluid.

## Conclusions

In this study, water-soluble nanographite was prepared through *in situ* emulsion polymerization. The graphite particles could disperse uniformly and steadily in aqueous environment after surface modification. The nanographite additive improved the friction-reducing and antiwear properties of the water-based cutting fluid. The mean friction coefficient and WSD reduced by 44% (from 0.106 to 0.059) and 49% (from 1.27 to 0.65 mm), respectively. The *P*_*B*_ value increased from 784 to 883 N. Meanwhile, the small surface tension indicated the enhancement of wettability. In general, nanographite additive made up the defect of current water-based cutting fluid whose lubrication ability was not ideal.

## Competing interests

The authors declare that they have no competing interests.

## Authors’ contributions

QC designed and carried out the experiment of nanographite hydrophilic modification, analyzed the data, and drafted the manuscript. XW and YL were mainly responsible for the preparation of water-soluble nanographite, and TY carried out the evaluation of lubrication performance. ZW supervised the research work and helped amend the manuscript. All authors read and approved the final manuscript.

## Authors’ information

QC, XW, YL, and TY are graduate students, and ZW is a professor at the College of Science in China University of Petroleum (East China).
